# 584 Where Are They Now? - A Single-Center Survey of Burn Surgery Fellow Graduates

**DOI:** 10.1093/jbcr/irac012.212

**Published:** 2022-03-23

**Authors:** Justine Prophete, Daud Lodin, Jim Gallagher, Abraham Houng

**Affiliations:** Holmdel High School, Holmdel, New Jersey; Weill Cornell Medical College, New York, New York; none, Phoenicia, New York; Weill Cornell Medical College, New York, New York

## Abstract

**Introduction:**

Burn surgery encompasses different surgical disciplines such as general surgery, trauma, surgical critical care and plastic surgery. Those who apply to burn fellowship also have various interests. This study’s purpose was to discern the various career paths of burn fellowship graduates. It also investigated the frequency of specific chosen specialties and workplace settings. The results of the study could be used to modify current clinical burn fellowship curriculum.

**Methods:**

A survey was created using an internet tool and emailed to graduates of the clinical burn fellowship program from an American Burn Association verified burn center. The survey was sent to graduates of the last 10 years (2010-2020). The questions inquired about surgical field of work, board certification, additional training experience, work setting, and time since fellowship graduation. The participants’ responses were recorded anonymously to account for biases. The quantitative data was then collected for each question, and the data was calculated into percentages.

**Results:**

Of the possible 17 fellows who graduated in the past ten years, 11 responded to our survey (64.7%). The most common surgical field pursued after the fellowship was plastic surgery with 5 graduates (45.5%). The following three fields all had 2 graduates each: burn surgery (burn/trauma), general surgery and wound care (18.2%). Almost all of the participants had completed additional training: 6 in plastic surgery (54.5%), and 4 in surgical critical care (36.4%). The work settings were primarily academic institutions (45.5%), followed by office-based practices (35.4%), ambulatory surgery centers (9.1%), and community hospitals (9.1%). Of the participants, 4 are board-certified in their chosen field, and 5 are board-eligible. Only 3 graduates are affiliated with a burn center.

**Conclusions:**

The results of the study indicate the interests and career choices of previous burn fellows. This information can be used to modify burn fellowship curriculums to the needs and future career paths of the fellows. Traditional clinical burn fellowship programs emphasize burn critical care and acute burn surgery. Since some of the graduates are interested in plastic surgery, burn reconstruction could be added to the current curriculum. Plastic surgery faculty members could also serve as mentors to the fellows.

